# Neurofilament Light Chain Protein in Plasma and Extracellular Vesicles Is Associated with Minimal Hepatic Encephalopathy and Responses to Rifaximin Treatment in Cirrhotic Patients

**DOI:** 10.3390/ijms241914727

**Published:** 2023-09-29

**Authors:** Alessandra Fiorillo, Juan José Gallego, Franc Casanova-Ferrer, Amparo Urios, María-Pilar Ballester, Teresa San Miguel, Javier Megías, Elena Kosenko, Joan Tosca, Maria-Pilar Rios, Desamparados Escudero-García, Carmina Montoliu

**Affiliations:** 1 Fundación de Investigación, Hospital Clínico Universitario de Valencia-INCLIVA, 46010 Valencia, Spain; alessa.fiorillo@gmail.com (A.F.); juanjo26.gr@gmail.com (J.J.G.); franc@alumni.uv.es (F.C.-F.); amparo.urios@uv.es (A.U.); 2 Servicio de Medicina Digestiva, Hospital Clínico Universitario de Valencia, 46010 Valencia, Spain; mapibafe@gmail.com (M.-P.B.); joantosca@gmail.com (J.T.); m.desamparados.escudero@uv.es (D.E.-G.); 3 Departamento de Patología, Facultad de Medicina, Universidad de Valencia, 46010 Valencia, Spain; teresa.miguel@uv.es (T.S.M.); javier.megias@uv.es (J.M.); 4 Institute of Theoretical and Experimental Biophysics, Russian Academy of Sciences, 142290 Pushchino, Russia; eakos@rambler.ru; 5 Servicio de Digestivo, Hospital Arnau de Vilanova, 46015 Valencia, Spain; mriosp73@hotmail.com; 6 Departamento de Medicina, Facultad de Medicina, Universidad de Valencia, 46010 Valencia, Spain

**Keywords:** neurofilament light chain protein, extracellular vesicles, minimal hepatic encephalopathy, rifaximin, inflammation, ammonia

## Abstract

Neurofilament light chain protein (NfL) levels reflect neuronal damage in several neurological diseases and have been proposed as a possible biomarker. Plasma extracellular vesicles (EVs) could play an important role as mediators of the inflammatory changes associated with inducing minimal hepatic encephalopathy (MHE) in cirrhotic patients. This study investigated the association of NfL levels in plasma and EVs with the presence of MHE in cirrhotic patients, and with responses to rifaximin treatment. The NfL levels in plasma and EVs were assessed in 71 patients with liver cirrhosis (40 with MHE and 31 without MHE) and 26 controls. A total of 31 patients with MHE received rifaximin treatment. We examined changes in NfL levels in plasma and EVs before and after 6 months of rifaximin treatment. The NfL measures were correlated with cognitive alterations and plasma inflammatory cytokines. MHE patients showed increased plasma levels of NfL, which were reverted after rifaximin treatment in patients who responded to treatment. The NfL content in EVs also showed a reversal pattern in MHE patients treated with rifaximin. In multivariable analyses, NfL levels were independently associated with the presence of MHE. We also showed that patients with high levels of both ammonia and fractalkine had significantly higher NfL levels than patients with low levels of least one of these parameters. Rifaximin treatment in MHE patients showed promising results in improving axonal damage, suggesting that rifaximin may have therapeutic benefits against disease progression in MHE.

## 1. Introduction

Minimal hepatic encephalopathy (MHE) is the earliest form of hepatic encephalopathy (HE) and can affect up to 80% of cirrhotic patients. MHE is characterized by attention deficits, psychomotor slowing, and mild cognitive impairment, which reduce the quality and length of life, but the disease lacks obvious clinical manifestations [1,2]. The gold standard for MHE diagnosis is the Psychometric Hepatic Encephalopathy Score (PHES), a psychometric battery that evaluates different cognitive and motor functions [3,4,5]. However, the PHES is time consuming and not widely used in daily clinical practice. Therefore, the identification of a biomarker that simplified the early diagnosis of MHE would lead to improved treatment and quality of life in patients with this disease.

Cirrhotic patients with MHE may present specific changes in peripheral inflammation and immunophenotype, particularly with the activation of Th22, Tfh, CD4^+^CD28^−^, and B lymphocytes [6]. Rifaximin, a poorly absorbable antibiotic, is well known for improving MHE symptoms by reducing ammonia levels and inflammatory parameters and improving psychometric test scores and quality of life [7,8,9].

Recently, neurofilament light chain protein (NfL) has been proposed as a possible biomarker in neurological diseases. NfL is a neuronal cytoplasmic protein responsible for maintaining the cytoskeleton of axons. Its levels in both cerebrospinal fluid (CSF) and blood proportionally reflect neuronal damage in a variety of neurological disorders, including inflammatory, neurodegenerative, traumatic, and cerebrovascular diseases [10,11,12]. Furthermore, plasma NfL levels could be a promising biomarker for distinguishing neurodegeneration and cognitive decline due to Alzheimer’s disease from other conditions that potentially cause cognitive impairment in the prodromal stages [13]. Recent studies have shown a possible association between neuroaxonal damage and blood NfL levels in patients with cirrhosis and MHE [14]. 

Nowadays, the importance of extracellular vesicles (EVs) and their role in physiological and pathological processes are increasingly recognized and supported. EVs are released from most cells, including neurons, and could serve as a biomarker for neurodegenerative diseases [15]. In this regard, plasma EVs could play an important role in the induction of specific immunophenotypic changes associated with MHE development in patients with liver cirrhosis [16].

The aim of this work was to evaluate a possible association between the occurrence and severity of MHE and the concentration of NfL in plasma and EV samples, both before and after treatment with rifaximin, thus identifying a potential future candidate biomarker for MHE diagnosis and response to rifaximin therapy.

## 2. Results

### 2.1. Study Population

The characteristics of the study participants are shown in Table 1. There were no differences in age between the control, MHE, and NMHE groups, but non-responding patients were significantly older than NMHE and responding patients (*p* < 0.01) (Table 1). There were no differences between NMHE and MHE patients in cirrhosis severity scales (Child–Pugh and MELD scores). However, both groups of patients had higher levels of liver damage markers, such as ALT and AST, and lower platelet counts than the control group (Table 1). MHE patients showed significantly lower albumin and higher bilirubin blood levels than the controls (Table 1). The participants’ performance in several psychometric tests, and their plasma levels of inflammatory parameters, are shown in Tables S1 and S2, respectively.

### 2.2. NfL Levels in Plasma and EVs

**Figure 1:**
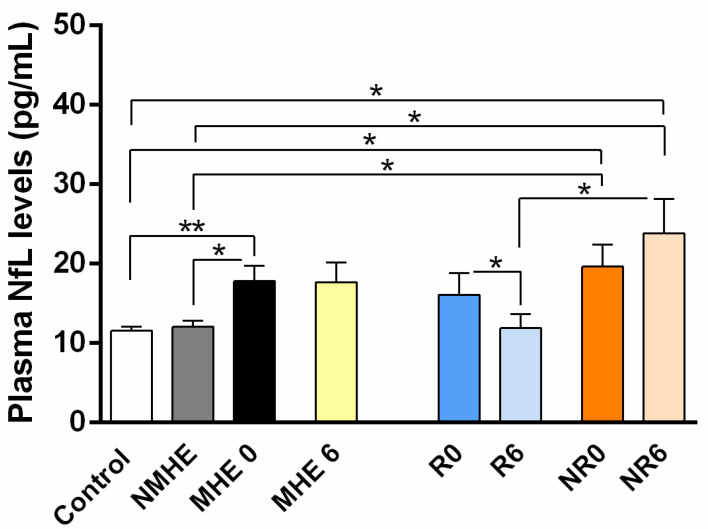
The plasma NfL levels in the controls and cirrhotic patients. The values are the mean ± SEM of the following groups: control n = 19, NMHE n = 31, MHE 0/6 n = 31, R0 and R6 n = 16, NR0 and NR6 n = 15. Values significantly different between groups are indicated by an asterisk (*) (* *p* < 0.05; ** *p* < 0.01). NMHE, patients without minimal hepatic encephalopathy; MHE 0/6, patients with minimal hepatic encephalopathy before and after treatment; R0/R6, responders before and after treatment; NR0/NR6, non-responders before and after treatment.

Patients with MHE had significantly higher plasma NfL levels than patients without MHE (NMHE) and controls (*p* < 0.01 and *p* < 0.05, respectively) (Figure 1, Table 2). No significant differences were found between the plasma NfL concentration of patients with MHE before and after six months of rifaximin treatment (Figure 1, Table 2). Nonetheless, when considering the responding and non-responding groups separately, we can see that responding patients showed a significant reduction in plasma NfL levels after treatment (R6) compared to the baseline (*p* < 0.05) (Figure 1, Table 2). In contrast, non-responding patients did not reduce the plasma concentrations of NfL protein after treatment (NR6), with significant differences compared to the responder (R6) (*p* < 0.05), NMHE, and control groups (*p* < 0.05) (Figure 1, Table 2). Interestingly, before treatment, responding patients (R0) had lower NfL levels than non-responding patients (NR0), although the difference did not reach significance. Furthermore, only the NR0 group showed significant differences in plasma NfL levels compared to those without MHE and the control group (*p* < 0.05) (Figure 1, Table 2). 

**Figure 2:**
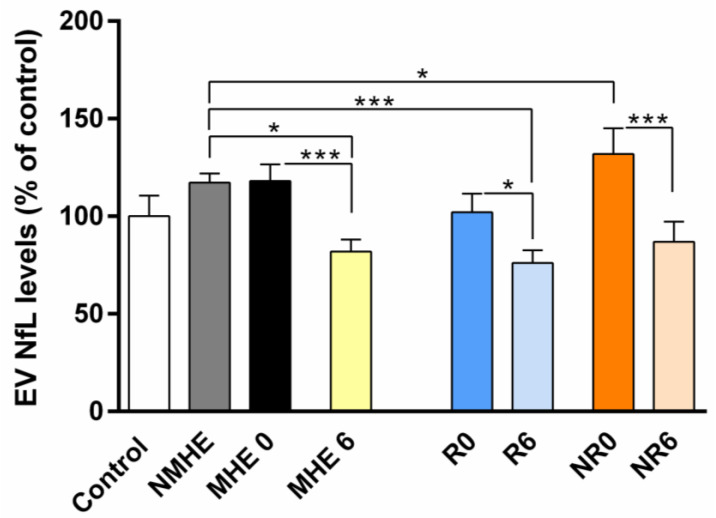
The NfL levels in EVs from the controls and cirrhotic patients. The values are expressed as the percentage of the control group and are the mean ± SEM. Control n = 10, NMHE n = 12, MHE 0/6 n = 29, R0 and R6 n = 14, NR0 and NR6 n = 15. Values significantly different between groups are indicated by an asterisk (*) (* *p* < 0.05; *** *p* < 0.001). NMHE, patients without minimal hepatic encephalopathy; MHE 0/6, patients with minimal hepatic encephalopathy before and after treatment; R0/R6, responders before and after treatment; NR0/NR6, non-responders before and after treatment.

In contrast, the NfL levels in EVs from patients with cirrhosis (with and without MHE) were increased, although there were no significant differences between the three study groups (Figure 2, Table 2). Interestingly, the NfL levels in EVs significantly decreased in MHE patients after rifaximin treatment (*p* < 0.001), with significantly lower levels than those in NMHE patients (*p* < 0.05) (Figure 2, Table 2). These reduced NfL levels in EVs after treatment were found in both the responding and the non-responding group, with significant differences compared to the baseline (*p* < 0.05 and *p* < 0.001, respectively) (Figure 2, Table 2). The NfL concentration in EVs, showed a trend of increasing in NR0 compared to R0 patients (*p* = 0.08).

The NfL levels in EVs after rifaximin treatment were also lower in both the responding and the non-responding group than in NMHE patients (*p* < 0.001 and *p* < 0.05, respectively) (Figure 2, Table 2).

### 2.3. Correlation of Psychometric Tests and Inflammatory Parameters with NfL Levels in Plasma and EVs in Cirrhotic Patients

As shown in Table 3, considering all the patient groups, we can see that there were significant negative correlations between plasma NfL levels and performance in most psychometric tests, and significant positive correlations with pro-inflammatory parameters. It is also notable that a higher plasma concentration of the NfL protein correlated with a lower PHES score (−0.458; *p* < 0.0001) (Table 3). Overall, patients without MHE showed no significant correlations between NfL concentration and the PHES or other psychometric tests. Separating MHE patients based on their rifaximin response, we observed that NR0 patients showed more significant correlations with psychometric tests and inflammatory parameters than R0 patients (Table 3). Moreover, a higher plasma NfL concentration correlated with higher ammonia levels in the group of all cirrhotic patients and in MHE and NR0 patients, whereas no significant correlation was reported in the NMHE or the R0 group (Table 3).

In cirrhotic patients with MHE, elevated plasma NfL levels correlated with increased levels of NfL in EVs. The correlations between the NfL levels in EVs and psychometric tests and inflammatory parameters are shown in Table 3. In MHE patients and the NR0 group, higher NfL levels in EVs correlated with a poorer performance in several d2 test parameters and, therefore, with a deficit in selective and sustained attention (Table 3). Overall, cirrhotic patients with MHE showed a significant correlation between NfL levels in EVs and several inflammatory parameters (Table 3).

### 2.4. Correlation Analysis with PHES Score and Potential Interplay between Hyperammonemia and Inflammation on Plasma NfL Levels

Several immunological and inflammatory parameters were altered in cirrhotic patients with MHE.

As shown in Table 4, significant correlations were found between the plasma levels of fractalkine (CX3CL1), ammonia and NfL, and the PHES, the score used to diagnose MHE (Table 4). In addition, we analyzed the possible diagnostic utility of these parameters in detecting the presence of MHE by performing an ROC analysis (Table 4). The plasma NfL and ammonia showed a similar AUC in the ROC analysis, with an AUC of 0.666 and 0.670, respectively, whereas the AUC for CX3CL1 was 0.860 (Table 4).

Regarding the plasma levels of NfL, at the cutoff of 12.6 pg/mL, the specificity was 68% and the sensitivity was 61%, while, for ammonia, at the cutoff of 18.2 μM, the specificity was 69% and the sensitivity was 70%. Finally, CX3CL1, at the cutoff of 504 pg/mL, showed a specificity and a sensitivity for MHE detection of 89% and 74%, respectively (Table 4).

**Figure 3:**
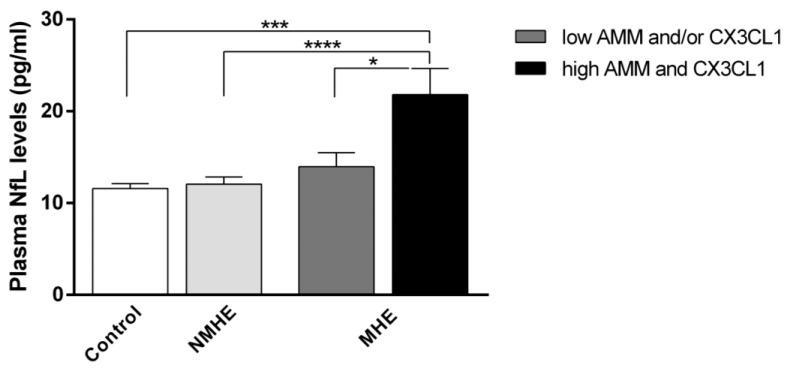
The plasma NfL levels in the controls and the NMHE and MHE patients, separated according to their ammonia and CX3CL1 levels. The values are the mean ± SEM of the following groups: control n = 19, NMHE n = 31, MHE with low ammonia and/or CX3CL1 n = 15, MHE with both high ammonia and CX3CL1 n = 21. Values significantly different between groups are indicated by an asterisk (*) (* *p* < 0.05; *** *p* < 0.001; **** *p* < 0.0001). NMHE, patients without minimal hepatic encephalopathy; MHE, patients with minimal hepatic encephalopathy; AMM: ammonia; CX3CL1, C-X3-C motif chemokine ligand 1.

In light of prior studies that have illustrated the potential interplay between hyperammonemia and inflammation as a key factor in the development of MHE [17,18], our objective was to explore the impact of hyperammonemia and heightened inflammation on plasma NfL levels in MHE patients. In this regard, our patient group was stratified according to their plasma levels of ammonia and CX3CL1, using the corresponding cutoff values extrapolated from the ROC curve analyses as a reference. Specifically, we categorized patients into two groups: those with low levels in least one parameter, and those with elevated levels in both parameters. Cirrhotic patients with MHE and with higher ammonia and CX3CL1 concentrations had significantly higher plasma NfL levels than MHE patients without this condition (MHE with high ammonia and CX3CL1, 21.82 ± 2.83 vs. control, 11.58 ± 0.52, *p* < 0.001; vs. NMHE, 12.06 ± 0.78, *p* < 0.0001; vs. MHE with low ammonia and/or CX3CL1, 13.95 ± 2.84, *p* < 0.05) (Figure 3).

### 2.5. Logistic Regression Analyses of Predictors of MHE Presence in Patients with Liver Cirrhosis

In our univariate analysis, including NFL levels and commonly available clinical parameters, MHE was significantly associated with the plasma levels of ammonia and NfL (Table 5). Multivariate logistic regression analysis, using the presence of MHE as the dependent variable and significant parameters in univariate analysis as independent variables, showed that only the NfL levels were significantly associated with MHE in patients with cirrhosis (OR: 1.105; CI, 1.007–1.213; *p* = 0.034) (Table 5).

## 3. Discussion

Based on this study, we confirmed that plasma NfL levels were significantly increased in patients with MHE and serve as an independent predictive parameter for MHE in cirrhotic patients. Furthermore, we have demonstrated, for the first time, that rifaximin reduces plasma NfL levels in patients who have successfully responded to treatment. 

An increase in NfL levels, both in blood and in CSF, was observed in various neurodegenerative conditions [10,11,12,13], and an increase in serum NfL levels was recently reported in cirrhotic patients with MHE [14].

The NfL levels in the plasma from MHE patients who responded to rifaximin treatment decreased significantly compared to the pre-treatment concentrations. In non-responding MHE patients, however, NfL levels increased significantly. These results are consistent with observations in a study of Alzheimer’s disease patients treated with rifaximin, confirming its clinical efficacy [19]. Some studies show sex differences in neurodegeneration and an association with the immune system. This association has been shown in neurological diseases such as Alzheimer’s disease, Parkinson’s disease, and amyotrophic lateral sclerosis [20]. However, regarding cognitive impairment following liver cirrhosis, there are still no definitive studies confirming how the sex differences relating to the immune system may influence the induction of MHE. In our study, the proportions between all the groups of cirrhotic patients are similar. Therefore, possible differences in the immune system due to sex cannot be affecting the comparisons between these groups.

In a previous study, our group demonstrated that rifaximin treatment reduced several inflammatory parameters and levels of the early activation marker CD69 in CD4^+^ T cells in responders; low cytokine levels were reduced in non-responders [9]. This study suggested that responding patients could be in an early stage of the inflammatory process, where rifaximin would be able to reverse immune alterations. The reduction in NfL in responding patients after rifaximin treatment would be mediated via the reversion of the inflammatory environment, which could improve neuroinflammation and axonal injury.

Our patient cohort consists of individuals with different etiologies, including viral, non-alcoholic steatohepatitis (NASH), and a predominant group represented by patients with alcoholic etiology. From our analysis, it emerged that patients with alcoholic etiology had increased NfL levels, with a greater increase observed in patients with MHE. The effect of a history of alcohol abuse on NfL should be further investigated in future studies to gain a deeper insight (Table S3).

We analyzed the correlations between plasma NfL levels and test performance, as well as the inflammatory parameters relevant to MHE, finding that the worse the performance in the PHES, the higher the plasma NfL levels in MHE patients; this was not observed in patients without cognitive impairment. Other studies have reported similar correlations between plasma NfL levels and cognitive and motor scores in other diseases such as Parkinson’s and Alzheimer’s diseases [21,22]. These findings suggest that plasma NfL levels are associated with MHE cognitive severity. Moreover, elevated plasma NfL levels correlate with deficits in attention, concentration, and motor coordination in patients with MHE. Interestingly, correlations with motor coordination were observed only in patients not responding to rifaximin treatment. These correlations reflect the relationship of poorer cognitive skills with high plasma NfL levels and greater axonal damage. In terms of inflammatory parameters, the plasma NfL levels correlate with the levels of ammonia and chemokines CCL20 and CX3CL1 in the MHE group and non-responding (NR0) patients at the baseline, who showed worse cognitive and motor states than the responders (R0). High ammonia, CCL20, and CX3CL1 levels are related to pathological mechanisms involving BBB permeability, immune cell infiltration, neuroinflammation, and cognitive and motor impairment in MHE [6]. Hence, elevated levels of these parameters would lead to axonal damage and higher NfL levels in the plasma of MHE patients. 

It is well known that in liver cirrhosis, ammonia metabolism is disrupted, causing hyperammonemia, which is a risk factor for MHE development [17,23]. Furthermore, in the present study, the increased plasma levels of NfL in patients with MHE correlated with higher concentrations of ammonia and several proinflammatory cytokines. Considering previous studies, which demonstrated that the combination of hyperammonemia and inflammation may be the key for the development of MHE [17,18], we aimed to investigate how hyperammonemia and high levels of inflammation may influence the plasma levels of NfL in MHE patients. 

In this regard, we found that plasma NfL, CX3CL1, and ammonia levels are good predictive parameters for identifying patients with MHE.

The NfL levels were significantly higher in patients who had high levels of both ammonia and CX3CL1 compared to the group with low levels of least one of these parameters. From this analysis, it can be confirmed that the development of MHE in cirrhotic patients requires a combination of both sufficiently high levels of ammonia and inflammation, which leads to an increase in plasma NfL levels.

It is known that the onset of MHE is linked to increased levels of ammonia and inflammation [17,18], which can be caused by intestinal dysbiosis [24]. Several studies have shown that rifaximin is able to alter gene expression that may be related to ammonia and inflammation levels [9,25,26]. Regarding ammonia levels, de Wit et al. conducted a study showing that rifaximin altered the expression of 131 genes in intestinal cells [25]. They found that rifaximin could alter the expression of genes related to nitrogen metabolism, such as glutaminase-2 and asparagine synthetase. These changes in nitrogen metabolism could lead to a reduction in ammonia levels and, thus, an improvement in the pathophysiology of MHE.

Inflammation levels are also affected by rifaximin treatment. Rifaximin, by inducing the expression of pregnane X receptors (PXRs), may also promote the transcription of genes for the detoxification of enzymes and cytokines [9,26], ultimately reducing inflammation and improving MHE. We also showed that rifaximin treatment reduced the expression of transcription factors related to helper T lymphocytes in MHE patients [9]. The results showed a reduction in the transcription factor RORC in all patients and a reduction in the transcription factors AHR and BCL6 in responders. The effects of rifaximin on the expression of these transcription factors could favor a reduction in inflammation, associated with an improvement in cognitive impairment in responder patients.

To improve MHE diagnosis, we performed univariate and multivariate analyses to identify the parameters that could predict MHE occurrence, including NfL levels and commonly available clinical parameters. In the multivariate analysis, only high NfL levels were predictive for the diagnosis of MHE in cirrhotic patients. 

Recent studies have pointed to the potential of EVs as biomarkers for neurological diseases, due to the possibility of analyzing neuron-derived EVs from blood samples [27]. EVs that contain NfL are derived from neurons, as the expression of this molecule is restricted to this cell type. In a preliminary analysis, we examined NfL levels in the total number of EVs isolated from plasma and found a slight, but not statistically significant rise in NfL levels in EVs from cirrhotic patients compared to the controls. Previous studies have reported an increase in the content of neuronal damage-related proteins, including NfL, in neuron-derived EVs from patients with neurological impairment. However, this increase was not observed when analyzing the total EVs isolated from plasma [28]. This suggests that the specific analysis of neuron-derived EVs, specifically targeting NfL, would enhance their utility as a biomarker, because neuron-derived EVs may provide a more accurate reflection of neuronal damage and serve as a more sensitive biomarker for neurological conditions. Our future studies will analyze the content of NfL in neuron-derived EVs within our study groups. The discrepancy between the levels of free NfL and NfL content in EVs could be attributed to the fact that the free NfL detected in plasma originates from end-stage neuronal damage, where neuronal death has occurred. In contrast, the NfL content in EVs may stem from neurons still in a senescent state, characterized by axonal cytoskeleton degradation but with ongoing neuronal activity. Rifaximin treatment can reduce the NfL levels in EVs isolated from plasma. This could be due to a reduction in EVs derived from neurons, resulting from the effect of rifaximin on glutamatergic neurotransmission. Neural EV release is partly regulated by glutamatergic activity, specifically through AMPA and NMDA receptors [29], which are altered in animal models of MHE [30,31]. Studies in animal models have demonstrated that liver damage induces neuroinflammation and alterations in NMDA receptors, alterations that rifaximin treatment was able to restore [32]. Other studies conducted on Alzheimer’s patients treated with rifaximin showed that microbiome modulation by rifaximin is associated with an improvement in neurodegenerative markers such as NfL, pTau, and GFAP [19]. Therefore, the reduction in NfL levels in plasma EVs could be attributed to the rifaximin effect of reducing neuroinflammation, leading to improved neuronal function and reduced EV release by neurons. As a result, the levels of NfL in plasma EVs are reduced. If we assume that the NfL in isolated EVs from plasma originates from senescent neurons, we could hypothesize that the effects of rifaximin improve the physiology of these neurons, although this may not halt the neurodegenerative process in patients who do not respond to rifaximin treatment. This impact on the neuronal physiology could explain the beneficial effects of rifaximin in the prevention of HE episodes and relapses [33,34].

In this initial analysis, our study was restricted to examining NfL levels in EVs isolated from plasma, with a limited number of subjects available. Future studies with a larger sample size will focus on analyzing the NfL content specifically in EVs derived from neurons in these patients.

Previous clinical trials investigating the effects of rifaximin on patients with cirrhosis and MHE, with an 8-week follow-up, have suggested that rifaximin has a positive impact on driving performance, cognitive abilities, quality of life, and reduced endotoxemia in patients with MHE [35,36]. Our results indicate that, following treatment with rifaximin, patients with MHE show a partial or total improvement in cognitive impairment associated with lower plasma levels of NfL, suggesting an enhancement in neuronal function in neurons undergoing decline. This implies that rifaximin may have a beneficial effect against disease progression in these patients. Incorporating the analysis of plasma NfL levels would assist in indirectly monitoring the neural function of the patients included in the clinical trials, as well as assessing its utility as a biomarker of a successful response to rifaximin treatment.

In conclusion, rifaximin administration in MHE patients is important for improving cognitive impairment. Although this is an observational study, our results suggest that changes induced by rifaximin treatment would lead to a decrease in plasma NfL levels in responding patients. This would indicate an indirect effect of rifaximin on the CNS, possibly slowing down axonal damage in patients who respond to treatment. Similarly, the changes produced by rifaximin could lead to a decrease in NfL content in EVs in all patients, which could indicate an improvement in overall neuronal function. This suggests that rifaximin could have a beneficial effect against disease progression in these patients. However, additional research and clinical trials are needed to further validate these findings and definitively establish the effectiveness of rifaximin in treating MHE.

## 4. Materials and Methods

### 4.1. Patients and Controls

Seventy-one patients with liver cirrhosis were recruited from the outpatient clinics at the Clínico and Arnau de Vilanova Hospitals of Valencia, Spain, from February 2014 to April 2023. The inclusion criteria were patients older than 18 years and a diagnosis of liver cirrhosis of any etiology based on liver histology or a combination of characteristic clinical, biochemical, and imaging features. The exclusion criteria were overt HE, recent alcohol intake (within the past 6 months), established neurological or psychiatric disorders, the recent use of drugs affecting cognitive function (within the past 6 weeks), hepatocellular carcinoma, and liver-related complications (including new-onset ascites, variceal bleeding, or infection requiring antibiotics) within the past 6 weeks. The patients included in the study did not show fever or any clinical or biological sign of recent infection. Twenty-six healthy volunteers (the control group) were also enrolled in the study once liver disease was ruled out via clinical, analytical, and serological tests. All the participants included in the study signed written informed consent. The patients and controls underwent clinical evaluation, psychometric tests, and blood analyses to determine ammonia levels and biochemical measurements on the same day. The study protocols were approved by the Scientific and Ethics Committees of both hospitals. The procedures followed were in accordance with the ethical guidelines of the Declaration of Helsinki.

### 4.2. Diagnosis of MHE, Psychometric Tests, and Rifaximin Treatment

Forty patients were diagnosed with MHE using the PHES [3]. The scores were adjusted for age and education level using Spanish normality tables (www.redeh.org/TEST_phes.htm accessed on 12 April 2023). Patients were classified as having MHE when the score was ≤−4 points. Healthy volunteers also undertook the PHES to rule out any kind of cognitive impairment. The other psychometric tests performed to study specific cognitive and motor alterations were the same as in [37]. Overall, 31 of 40 MHE patients were treated with rifaximin (1.2 g/day, in three doses of 400 mg every 8 h) for 6 months. Patients treated with rifaximin for 6 months in whom MHE improved (a PHES > −4 or an improved PHES ≥ 4 points) were considered responders (R0 before treatment, R6 after treatment), while patients who, even after treatment, continued to exhibit MHE (a PHES ≤ −4 or an improved PHES < 4 points) were considered non-responders (NR0 before treatment, NR6 after treatment). Blood collection and psychometric tests were performed after 6 months of treatment. 

### 4.3. Laboratory Measurements in Blood Samples

The blood ammonia was measured immediately after blood extraction with the Ammonia Test Kit II for the PocketChem BA system (Arkray, Inc., Kyoto, Japan) according to the manufacturer’s instructions. The blood samples were centrifuged for 10 min at 1500× *g*, and the plasma was immediately separated and kept at −80 °C for subsequent cytokine analysis and EV isolation. The concentrations of IL-6, IL-18, IL-13, TGF-β, IL-22, CCL2, CCL20, and CX3CL1 (R&D Systems, Minneapolis, MN, USA) were measured via ELISA according to the manufacturer’s instructions.

### 4.4. EV Isolation

EVs were isolated from plasma via size-exclusion chromatography, using qEV 2/70 nm columns from IZON (Izon, Lyon, France), as in [16]. The presence of EVs in samples was determined and probed for purity via transmission electron microscopy, nanoparticle tracking analysis and the analysis of EV markers, and the absence of endoplasmic reticulum markers via Western blotting, as in [16]. To lyse the EVs, a volume corresponding to 40 µg was mixed with the same volume of M-PER Reagent (M-PER Mammalian Protein Extraction Reagent; ThermoFisher, Waltham, MA, United States) containing a protease and phosphatase inhibitor (Pierce™ Protease and Phosphatase Inhibitor Mini Tablets; ThermoFisher) for 30 min on ice, then the samples were centrifuged at 4000 rpm for 5 min at 4 °C, and the supernatant was used for protein analysis.

### 4.5. NFL Measurement in Plasma and EV Samples

The NFL concentrations in the plasma and EV samples were analyzed via the NF-light Advantage Assay using SIMOA™ HD-X equipment (Quanterix Corp., Billerica, MA, USA) according to the manufacturer’s instructions. The EV samples were diluted to 200 µL with a sample dilution kit.

### 4.6. Statistical Analysis

Continuous variables were reported as mean and standard error of the mean (SEM) and comparisons were performed using Student’s *t*-test or one-way analysis of variance (ANOVA) followed by a post hoc Tukey’s multiple comparison test. Categorical data were analyzed via the chi-square test. Bivariate correlations were evaluated using Spearman’s rho correlation test. Univariate and multivariate logistic regressions were performed using MHE as the dependent variable. The potential explanatory variables used in the univariate analysis were those showing significant (*p* < 0.05) differences between NMHE and MHE patients. Multivariate logistic regression analysis was performed, including as independent variables those that were significant in the univariate analysis. Receiver operating characteristic (ROC) curves were performed to determine the sensitivity and specificity of the predictor variables found. The results were analyzed with GraphPad PRISM vs. 8 (GraphPad Software; San Diego, CA, USA) and SPSS vs. 28.0 (SPSS Inc., Chicago, IL, USA). The probability level accepted for significance was *p* < 0.05.

## Figures and Tables

**Table 1:** The clinical and demographic variables of the participants.

Variables	Control (n = 26)	Cirrhotic Patients		MHE Patients Treated with Rifaximin (Before Treatment)	
		NMHE (n = 31)	MHE (n = 40)	R0 (n = 16)	NR0 (n = 15)
Sex, n (%)					
Male	14 (54)	27 (87)	31 (78)	13 (81)	12 (80)
Female	12 (46)	4 (13)	9 (22)	3 (19)	3 (20)
Age (years) ^a^	61.7 ± 1.1	60.3 ± 1.4	63.5 ± 1.2	59.3 ± 1.6	67.1 ± 1.7 ^αα/ββ^
Etiology of cirrhosis, n (%)					
Alcohol	-	19 (61)	20 (50)	11 (69)	6 (40)
HBV/HCV	-	7 (23)	9 (22)	3 (19)	4 (27)
NASH	-	2 (6)	8 (20)	1 (6)	5 (33)
Others	-	3 (10)	3 (8)	1 (6)	0 (0)
Child–Pugh score (A/B/C)	-	24/5/2	25/13/2	10/6/0	9/5/1
MELD score ^a^	-	8.8 ± 0.5	9.9 ± 0.6	9.5 ± 0.5	9.9 ± 1.3
Clinical parameters					
Ammonia (µM) ^a^	10.7 ± 1.0	23.0 ± 4.5 *	40.2 ± 5.2 ***^/α^	45.6 ± 10.5	32.5 ± 6.8
AST (U/L) ^a^	23.8 ± 1.4	36.1 ± 3.3 **	45.3 ± 5.6 **	42.9 ± 5.7 *	35 ± 5.1
ALT (U/L) ^a^	24.2 ± 1.5	29.1 ± 1.9	34.1 ± 3.9	33.6 ± 4.3	27.3 ± 2.2
GGT (U/L) ^a^	30.4 ± 5.2	71.6 ± 14.1 *	79.6 ± 9.8 ***	83.9 ± 13.9 **	73.5 ± 16.3
Albumin (g/dL) ^a^	4.4 ± 0.1	4.0 ± 0.1	3.7 ± 0.1 ***	3.7 ± 0.1 ***	3.9 ± 0.1 **
Bilirubin (mg/dL) ^a^	0.58 ± 0.06	1.26 ± 0.19	1.64 ± 0.3 *	1.44 ± 0.25 *	1.85 ± 0.74
Creatinine (mg/dL) ^a^	0.84 ± 0.04	0.87 ± 0.07	0.85 ± 0.04	0.79 ± 0.05	0.94 ± 0.06
Platelets (·10^9^/L) ^a^	237.1 ± 15.5	135.1 ± 12.8 ***	125.1 ± 9.9 ***	105.3 ± 10.3 ***	131.1 ± 15.1 ***

^a^ Values are the mean ± SEM. Between-group comparisons were performed using ANOVA, followed by a post hoc Tukey’s test for continuous data and chi-square (*χ*^2^) test for categorical data. Values significantly different from those in the controls are indicated by an asterisk (*), from those in NMHE patients by α, and from those in responding patients by β (*^/α^ *p* < 0.05; **^/αα/ββ^ *p* < 0.01; *** *p* < 0.001). NMHE, patients without minimal hepatic encephalopathy; MHE, patients with minimal hepatic encephalopathy; R0, responders and NR0, non-responders before treatment; HBV, hepatitis B virus; HCV, hepatitis C virus; NASH, non-alcoholic steatohepatitis; MELD, Model for End-stage Liver Disease; AST, aspartate transaminase; ALT, alanine transaminase; GGT, gamma-glutamyl transferase.

**Table 2:** The plasma and EV NfL levels in control subjects and cirrhotic patients at the baseline and follow-up according to their response to rifaximin treatment.

NfL Levels	Control	Cirrhotic Patients		MHE Patients Treated with Rifaximin			
		NMHE	MHE	R0	R6	NR0	NR6
Plasma ^a^	11.6 ± 0.5	12.1 ± 0.8	17.8 ± 1.9 **^/α^	16.1 ± 2.7	11.9 ± 1.8 ^δ^	19.6 ± 2.8 *^/α^	23.9 ± 4.3 *^/α/β^
EVs ^b^	100.2 ± 10.3	117.2 ± 4.7	118.0 ± 8.6	102.1 ± 9.4	76.2 ± 6.43 ^ααα/δ^	131.9 ± 13.12	86.8 ± 10.5 ^α/δδδ^

The values are the mean ± SEM. ^a^ pg/mL; ^b^ percentage of the control group. Values significantly different from the control are indicated by an asterisk (*), from NMHE patients by ^α^, from responders by ^β^, and from patients before treatment by ^δ^ (*^/α/β/δ^ *p* < 0.05; ** *p* < 0.01; ^ααα/δδδ^ *p* < 0.001). NMHE, patients without minimal hepatic encephalopathy; MHE, patients with minimal hepatic encephalopathy; R0/R6, responders before and after treatment; NR0/NR6, non-responders before and after treatment; NfL, neurofilament light chain; EVs, extracellular vesicles.

**Table 3:** The correlations between tests and inflammatory parameters and plasma and EV NfL levels in cirrhotic patients.

Correlations with Plasma NfL Levels										
Parameter	Cirrhotic Patients		NMHE Patients		MHE Patients		R0 Patients		NR0 Patients	
	Correlation Coefficient	*p* Value	Correlation Coefficient	*p* Value	Correlation Coefficient	*p* Value	Correlation Coefficient	*p* Value	Correlation Coefficient	*p*Value
PHES	−0.458	<0.0001			−0.510	0.001	−0.527	0.010	−0.642	0.010
**Stroop Test**										
Congruent	−0.314	0.010								
Neutral	−0.356	0.003								
Incongruent	−0.305	0.012								
**d2 Test**										
TR values	−0.467	<0.0001			−0.521	0.004			−0.720	0.013
TA values	−0.487	<0.0001			−0.567	0.001	−0.677	0.011	−0.656	0.029
TOT values	−0.524	<0.0001			−0.605	0.001	−0.648	0.017	−0.710	0.014
CON values	−0.503	<0.0001			−0.608	<0.0001	−0.763	0.002	−0.663	0.026
**Coordination tests**										
Bimanual	0.468	<0.0001			0.406	0.013			0.517	0.049
Visuomotor	0.557	<0.0001			0.513	0.001			0.848	<0.0001
**Oral SDMT test**										
Scaled score	−0.293	0.014								
**Biochemical parameters**										
Ammonia	0.320	0.008			0.363	0.030			0.529	0.042
IL-13	−0.468	0.014	−0.825	0.006						
CCL20	0.438	0.001			0.402	0.020			0.829	<0.0001
IL-22	0.259	0.046							0.695	0.026
CX3CL1	0.445	<0.0001			0.355	0.042				
NfL (EVs)	0.519	<0.0001			0.589	<0.0001				
**Correlations with NfL levels in EVs**										
**d2 Test**										
TR values	−0.385	0.011			−0.447	0.010			−0.733	0.007
TA values									−0.711	0.010
TOT values	−0.309	0.044							−0.659	0.020
**Biochemical parameters**										
IL-22	0.404	0.006	−0.569	0.042	0.486	0.005			0.539	0.047
CX3CL1	−0.392	0.015			−0.415	0.028				
NfL (plasma)	0.519	0.000			0.589	<0.0001			0.562	0.023

The correlation coefficient and *p* value for Pearson correlations are shown. NMHE, patients without minimal hepatic encephalopathy; MHE, patients with minimal hepatic encephalopathy; R0, responders before treatment; NR0, non-responders before treatment; PHES, psychometric hepatic encephalopathy score; SDMT, symbol digit modalities test (oral version); TRs, total responses; TAs, total right answers; TOT, total effectiveness of the test; CON, concentration index; IL, interleukin; CCL20, C-C motif chemokine ligand 20; CX3CL1, C-X3-C motif chemokine ligand 1 (fractalkine); NfL, neurofilament light chain; NfL(EVs); neurofilament light chain (extracellular vesicles).

**Table 4:** The correlations between the PHES score and inflammatory parameters and plasma NfL levels, and ROC analyses to assess the diagnostic capacity of these parameters to detect MHE.

	Correlation ^a^ with PHES Score		Receiver Operating Characteristic (ROC) Curves ^b^				
Parameters	Correlation Coefficient	*p* Value	AUROC (95% CI)	*p* Value	Cutoff	Sensitivity	Specificity
IL-6	0.013	0.945	0.463 (0.256–0.670)	0.751			
IL-18	−0.045	0.740	0.444 (0.294–0.594)	0.479			
IL-13	0.032	0.872	0.602 (0.332–0.873)	0.389			
CCL20	−0.136	0.332	0.558 (0.403–0.712)	0.486			
IL-22	−0.165	0.213	0.644 (0.500–0.788)	0.060			
TGF-β	−0.025	0.856	0.548 (0.394–0.701)	0.546			
CCL2	−0.176	0.190	0.640 (0.495–0.784)	0.074			
**Ammonia**	**−0.270**	**0.025**	**0.670 (0.539–0.801)**	**0.016**	**18.2 ^c^**	**70**	**69**
**CX3CL1**	**−0.545**	**<0.0001**	**0.860 (0.769–0.951)**	**0.000**	**504 ^d^**	**74**	**89**
**NfL**	**−0.331**	**0.005**	**0.666 (0.539–0.792)**	**0.019**	**12.6 ^d^**	**61**	**68**

^a^ Spearman’s rho correlation between the parameters and the PHES score. ^b^ Receiver operating characteristic (ROC) curves in the diagnosis of minimal hepatic encephalopathy in the patient cohort. ^c^ µM; ^d^ pg/mL. AUROC, area under the receiver operating curve; CI, confidence interval; IL, interleukin; CCL20, C-C motif chemokine ligand 20; TGF-β, transforming growth factor-β; CX3CL1. C-X3-C motif chemokine ligand 1 (fractalkine); CCL2, C-C motif chemokine ligand 2, NfL, neurofilament light chain. In bold, variables that were significant (*p* < 0.05) in the analyses.

**Table 5:** Univariate and multivariate logistic regression analyses including NFL levels and commonly available clinical parameters to predict the presence of MHE in cirrhotic patients.

Univariate Logistic Regression Analyses		
Independent Variables	OR (95% CI)	*p* Value
AST	1.015 (0.991–1.039)	0.229
ALT	1.016 (0.984–1.048)	0.329
GGT	1.002 (0.993–1.011)	0.637
Albumin	0.430 (0.168–1.098)	0.078
Bilirubin	1.225 (0.806–1.861)	0.342
Creatinine	0.787 (0.168–3.691)	0.761
Platelets	0.998 (0.991–1.005)	0.525
MELD	1.124 (0.948–1.333)	0.179
**Ammonia**	**1.023 (1.002–1.045)**	**0.029**
**NfL**	**1.115 (1.022–1.217)**	**0.014**
**Multivariate Logistic Regression Analysis**		
**Independent Variables**	**OR (95% CI)**	***p* Value**
**NfL**	**1.105 (1.007–1.213)**	**0.034**
Ammonia	1.013 (0.991–1.035)	0.244

In both uni-and multivariate analyses, the dependent variable was the presence of MHE. In the multivariate analysis, the independent variables were those that were significant (*p* < 0.05) in the univariate analysis (in bold). AST, aspartate transaminase; ALT, alanine transaminase; GGT, gamma-glutamyl transferase; MELD, model end-stage liver disease; NfL, neurofilament light chain; OR, odds ratio; CI, confidence interval.

## Data Availability

The data are contained within the article and the Supplementary Materials.

## Supplementary Materials

The following supporting information can be downloaded at: https://www.mdpi.com/article/10.3390/ijms241914727/s1.
