# Supplementary material for: Neurofilament Light Chain Protein in Plasma and Extracellular Vesicles Is Associated with Minimal Hepatic Encephalopathy and Responses to Rifaximin Treatment in Cirrhotic Patients
Source: Int J Mol Sci. 2023 Sep 29;24(19):14727. doi: 10.3390/ijms241914727 (PMC10572420; doi:10.3390/ijms241914727)
Supplement: Supplementary file 1 [file ijms-24-14727-s001.zip › ijms-2620468-supplementary.pdf]

Table S1. Psychometric test results of control subjects and cirrhotic patients at baseline and follow-up according to response to rifaximin treatment.

Psychometric test	Control	Cirrhotic patients		MHE patients treated with rifaximin			
		NMHE	MHE	R0	R6	NR0	NR6
**PHES**	1.1 ± 0.3	-0.3 ± 0.2***	-7.4 ± 0.5$^{***/\alpha\alpha\alpha}$	-7.2 ± 0.9$^{***/\alpha\alpha\alpha}$	-3.5 ± 0.6$^{\delta\delta\delta}$	-7.7 ± 0.7$^{***/\alpha\alpha\alpha}$	-7,7 ± 0,8$^{\beta\beta\beta}$
**Stroop test**							
Congruent task	51.1 ± 2.1	45.3 ± 1.7	32.8 ± 1.5$^{***/\alpha\alpha\alpha}$	33.6 ± 2.6$^{***/\alpha\alpha}$	38.9 ± 2.8$^{\delta}$	33.3 ± 2.5$^{***/\alpha\alpha}$	31.8 ± 2.9$^{\beta}$
Neutral task	51.3 ± 1.8	43.7 ± 1.5**	33.9 ± 1.1$^{***/\alpha\alpha\alpha}$	34.3 ± 2$^{***/\alpha\alpha}$	39.2 ± 2.3$^{\delta}$	32.8 ± 1.6$^{***/\alpha\alpha\alpha}$	31.4 ± 2.2$^{\beta}$
Incongruent task	50.7 ± 2	43.9 ± 1.9*	34.8 ± 1.3$^{***/\alpha\alpha\alpha}$	33.3 ± 2.3$^{***/\alpha\alpha}$	40.4 ± 2.1$^{\delta}$	34 ± 2.1$^{***/\alpha\alpha}$	36.1 ± 3.1
**d2 test**							
TR	395.6 ± 15.7	330 ± 13.5**	257.4 ± 15.3$^{***/\alpha\alpha}$	283.1 ± 20.4***	317.1 ± 19.3$^{\delta}$	214.1 ± 25.4$^{***/\alpha\alpha\alpha/\beta}$	240 ± 21.9$^{\beta\beta}$
TA	145.1 ± 8.8	122.5 ± 6.8	87.4 ± 6.4$^{***/\alpha\alpha}$	94.4 ± 7.7**	114.4 ± 9$^{\delta}$	74.5 ± 12.9$^{***/\alpha\alpha}$	89.9 ± 10.8$^{\beta}$
O	15.2 ± 3.3	13.9 ± 2.1	22.1 ± 4	26.4 ± 7.2	18.5 ± 5.7	16.3 ± 4	11.6 ± 2.8
C	2.5 ± 0.9	4.2 ± 1.4	8.3 ± 2.4	9.2 ± 4.1	5.1 ± 2.8	11 ± 4.1	5.5 ± 2.4
TOT	372.2 ± 18.7	304.3 ± 15.2*	227.9 ± 14.9$^{***/\alpha\alpha}$	247.5 ± 18.7***	293.7 ± 19.4$^{\delta}$	189.2 ± 27.9$^{***/\alpha\alpha/\beta}$	222.9 ± 24.8$^{\beta}$
CON	145.6 ± 7.1	119 ± 7.3	79.1 ± 7.5$^{***/\alpha\alpha\alpha}$	85.2 ± 10.5$^{***/\alpha}$	109.3 ± 10.4$^{\delta}$	63.5 ± 14.3$^{***/\alpha\alpha\alpha}$	84.4 ± 12.4
**Oral SDMT Test**							
Correct pairing	51 ± 1.5	38.8 ± 1.6***	23.5 ± 1.8$^{***/\alpha\alpha\alpha}$	26.9 ± 3.1$^{***/\alpha\alpha\alpha}$	33.6 ± 2.4$^{\delta\delta}$	18.9 ± 2.4$^{***/\alpha\alpha\alpha/\beta}$	19.8 ± 2.4$^{\beta\beta\beta}$
Scaled score	13.3 ± 0.4	10.2 ± 0.4***	7.3 ± 0.4$^{***/\alpha\alpha\alpha}$	7.3 ± 0.8$^{***/\alpha\alpha\alpha}$	9.0 ± 0.5$^{\delta\delta}$	7.0 ± 0.6$^{***/\alpha\alpha\alpha}$	7.0 ± 0.6$^{\beta\beta}$
Errors pairing	0.5 ± 0.2	1.3 ± 0.3*	1.5 ± 0.3**	1.3 ± 0.3*	1.8 ± 0.4	1.9 ± 0.6	1.3 ± 0.4
Total pairing	47.2 ± 4.3	40.1 ± 1.6	25 ± 1.7$^{***/\alpha\alpha\alpha}$	28.2 ± 3.1$^{***/\alpha\alpha}$	35.4 ± 2.3$^{\delta\delta}$	20.8 ± 2.5$^{***/\alpha\alpha\alpha/\beta}$	21.1 ± 2.4$^{\beta\beta\beta}$
**Digit span test**							
Digits forward	9.9 ± 0.6	7.9 ± 0.4**	6.6 ± 0.3$^{***/\alpha}$	6.8 ± 0.3***	7.4 ± 0.6	6 ± 0.5$^{***/\alpha}$	6.7 ± 0.5
Digits backward	6.8 ± 0.5	4.5 ± 0.3***	3.9 ± 0.3***	3.9 ± 0.5***	5.2 ± 0.7$^{\delta}$	3.6 ± 0.3***	4 ± 0.4
Digits total	16.7 ± 0.9	12.4 ± 0.5***	10.5 ± 0.6***	10.8 ± 0.7***	12.6 ± 1.3$^{\delta}$	9.6 ± 0.7$^{***/\alpha}$	10.7 ± 0.8$^{\delta}$
Number-letter test	9.1 ± 0.6	7.4 ± 0.5	5.4 ± 0.5$^{***/\alpha\alpha}$	6.1 ± 0.8**	6.2 ± 0.9	4.4 ± 0.7$^{***/\alpha\alpha}$	4.6 ± 0.7
**Coordination test**							
Bimanual	2.1 ± 0.1	2.3 ± 0.1*	3.5 ± 0.2$^{***/\alpha\alpha\alpha}$	3.1 ± 0.2$^{**/\alpha\alpha}$	2.9 ± 0.2	3.9 ± 0.5$^{**/\alpha}$	3.6 ± 0.3$^{\beta}$
Visuo-motor	2.4 ± 0.1	2.8 ± 0.1***	4.0 ± 0.2$^{***/\alpha\alpha\alpha}$	3.5 ± 0.2$^{***/\alpha\alpha}$	3.4 ± 0.2	4.4 ± 0.4$^{***/\alpha\alpha/\beta}$	4.4 ± 0.4$^{\beta}$

Values are the mean ± SEM. Values significantly different from the control are indicated by an asterisk (*), from NMHE patients by $^{\alpha}$, from responder patients by $^{\beta}$ and from patients before treatment by $^{\delta}$ ($^{*/\alpha/\beta/\delta}$ $p<0.05$; $^{**/\alpha\alpha/\beta\beta/\delta\delta}$ $p<0.01$; $^{***/\alpha\alpha\alpha/\beta\beta\beta/\delta\delta\delta}$ $p<0.001$). NMHE, patients without minimal hepatic encephalopathy; MHE, patients with minimal hepatic encephalopathy; R0/R6, responder patients before and after treatment; NR0/NR6, non-responder patients before and after treatment; PHES, psychometric hepatic encephalopathy score; TR, total responses; TA, total right answer; O, omission errors; C, commission errors; TOT, effectiveness index; CON, concentration index; SDMT, symbol digit modalities test (oral version).

Table S2. Plasma inflammatory parameters of control subjects and cirrhotic patients at baseline and follow-up according to their response to rifaximin treatment.

Inflammatory parameters	Control	Cirrhotic patients		MHE patients treated with rifaximin			
		NMHE	MHE	R0	R6	NR0	NR6
**IL-6**[a]	1.3 ± 0.2	2.4 ± 0.3	2.9 ± 0.6	2.9 ± 0.5**	2.0 ± 0.4	3.2 ± 1.4	2.2 ± 1.4
**IL-18**[a]	197.7 ± 33.2	387.4 ± 36**	445 ± 72.5**	441.1 ± 83.3**	493.3 ± 91.9	503.1 ± 147.7	641.5 ± 289.6
**IL-13**[a]	1.4 ± 0.9	3.8 ± 1.6	3.3 ± 0.8	3.8 ± 1.6	3.1 ± 1.6	1.9 ± 0.4	1.4 ± 0.3
**CCL20**[a]	6.7 ± 1.4	20.1 ± 2.9**	39.3 ± 8.9**	32 ± 7.3*	38.4 ± 8.2	34.7 ± 13.8	23.5 ± 8.6
**IL-22**[a]	27.5 ± 2.9	34.8 ± 4.4	82.8 ± 15.1$^{**/\alpha}$	77.5 ± 17.1*	91.5 ± 16.1	101.2 ± 33.1	87.2 ± 17.5
**TGF-β**[b]	115.2 ± 24.9	96.5 ± 17.8	111.5 ± 17.1	114.0 ± 30.8	100.3 ± 44.8	117.2 ± 21.2	44.4 ± 15
**CX3CL1**[a]	305.2 ± 64.4	335.2 ± 30	716.8 ± 57.4$^{***/\alpha\alpha\alpha}$	750.7 ± 70.6$^{***/\alpha\alpha\alpha}$	726.6 ± 72	715 ± 107.5$^{*/\alpha}$	733.1 ± 121.6
**CCL2**[a]	11.6 ± 2.4	11.5 ± 2.1	54.4 ± 15.4$^{*/\alpha}$	65.1 ± 27.3	8.7 ± 1.7$^{\delta}$	51.7 ± 22.7	13.1 ± 2.9

Values are the mean ± SEM. [a] pg/mL; [b] ng/mL. Values significantly different from control are indicated by an asterisk (*), from NMHE patients by $^{\alpha}$ and from patients before treatment by $^{\delta}$ ($^{*/\alpha/\delta}$ $p<0.05$; ** $p<0.01$; $^{***/\alpha\alpha\alpha}$ $p<0.001$). NMHE, patients without minimal hepatic encephalopathy; MHE, patients with minimal hepatic encephalopathy; R0/R6, responders before and after treatment; NR0/NR6, non-responders before and after treatment; IL, interleukin; CCL20, C-C Motif Chemokine Ligand 20; TGF-β, transforming growth factor-β ; CX3CL1. C-X3-C motif chemokine ligand 1; CCL2, C-C motif chemokine ligand 2.

Table S3. Plasma NfL levels in control subjects and cirrhotic patients according to cirrhosis etiology.

	Control	Cirrhotic patients		
		HBV/HCV	Alcohol	NASH
**Total group**	11.6 ± 0.5	10.5 ± 0.9	15.2 ± 1.4$^{*/\varepsilon}$	19.5 ± 4.1
**NMHE**	-	9.2 ± 0.8	12.9 ± 1.0$^{\varepsilon}$	11.7 ± 4.4
**MHE**	-	11.4 ± 1.4	17.5 ± 2.5$^{*/\varepsilon}$	21.4 ± 4.9

Values are expressed in pg/ml and are the mean ± SEM. Values significantly different from the control are indicated by an asterisk (*) and from HBV/HCV etiology patients by $^{\varepsilon}$ ($^{*/\varepsilon}$ $p<0.05$). PHES, psychometric hepatic encephalopathy score; NMHE, patients without minimal hepatic encephalopathy; MHE, patients with minimal hepatic encephalopathy; HBV, hepatitis B virus; HCV, hepatitis C virus; NASH, non-alcoholic steatohepatitis.
